# Circadian Clock and Liver Cancer

**DOI:** 10.3390/cancers13143631

**Published:** 2021-07-20

**Authors:** María Crespo, Magdalena Leiva, Guadalupe Sabio

**Affiliations:** Centro Nacional de Investigaciones Cardiovasculares Carlos III, 28029 Madrid, Spain; maria.crespo@cnic.es

**Keywords:** circadian clock, liver, hepatocellular carcinoma

## Abstract

**Simple Summary:**

The circadian coordination of metabolism is tightly regulated, and its alteration can trigger several diseases, including liver steatohepatitis and cancer. Many factors (such as diet and jet lag) shape both the liver molecular clock and the circadian transcription/translation of genes related to different metabolic pathways. Here, we summarize our current knowledge about the molecular mechanisms that control this circadian regulation of liver metabolism.

**Abstract:**

Circadian clocks control several homeostatic processes in mammals through internal molecular mechanisms. Chronic perturbation of circadian rhythms is associated with metabolic diseases and increased cancer risk, including liver cancer. The hepatic physiology follows a daily rhythm, driven by clock genes that control the expression of several proteins involved in distinct metabolic pathways. Alteration of the liver clock results in metabolic disorders, such as non-alcoholic fatty liver diseases (NAFLD) and impaired glucose metabolism, that can trigger the activation of oncogenic pathways, inducing spontaneous hepatocarcinoma (HCC). In this review, we provide an overview of the role of the liver clock in the metabolic and oncogenic changes that lead to HCC and discuss new potentially useful targets for prevention and management of HCC.

## 1. Introduction to Circadian Rhythms

Circadian rhythms in mammals allow tissues to adapt to their biological function and to anticipate external changes, which leads to the synchronization of mammal physiology to the environment and to the 24-h solar day. These daily fluctuations in physiological and behavioral processes rely on an intrinsic molecular clock and its interaction with environmental changes. The external cues that dictate circadian behaviors are known as zeitgebers. The light/dark cycle is the most studied and common zeitgeber, but many other signals, including the feeding/fasting cycle, melatonin or body temperature, are considered zeitgebers as well [1]. Disruption of circadian rhythms in mammals can result in the development of different diseases, such as cancer and neurodegenerative and metabolic disorders. Therefore, it is fundamental to study the molecular mechanisms that sustain these circadian rhythms in order to better understand how disease can originate from abruptions in daily fluctuations.

The circadian system in mammals relies on the presence of a molecular clock in every organ and every cell of the body, thus generating multiple autonomous clocks. All of these self-sustaining clocks are synchronized by a central molecular clock within the suprachiasmatic nucleus (SCN), composing a hierarchically structured circadian system. This master synchronizer is located in the anteroventral hypothalamus, where it receives external photic information derived from the retina as well as non-photic information from the other tissues and organs of the body. The SCN integrates all of this information and generates output signals to the peripheral clocks, setting the basis for an entrainment between the tissues as well as to the 24-h day. There are three major sources of input signals arriving at the SCN: (i) the photic information comes from the transduction of the light by photosensitive retinal cells, which transmit this photic information to neurons in the SCN by releasing melanopsin; (ii) melanopsin then acts on another region of the brain, the intergeniculate leaflets (IGL), to instruct the release of neurotransmitters from these neurons to the SCN. These two mechanisms support both a direct and an indirect entrainment of SCN to light, with a delay between both inputs that makes the SCN response more precise and determinant; and (iii) non-photic information, such as that related to activity and exercise, arrive at the SCN from the median/dorsal raphe nucleus (MRN/DRN) [1].

Although peripheral clocks act with certain autonomy, their orchestration via the SCN is necessary to coordinate a network between peripheral clocks [2]. SCN coordinates peripheral clocks directly through humoral and synaptic signals. For instance, sympathetic denervation of some peripheral clocks results in altered circadian oscillations [3], and glucocorticoids are released in a circadian manner to phase-entrain peripheral oscillators [4]. Further, by coupling feeding–fasting and rest–activity cycles, the SCN can indirectly dictate daily fluctuations in peripheral tissues. Normally, direct and indirect pathways cooperate to entrain the peripheral timekeepers. However, time-restriction feeding is able to desynchronize peripheral clocks from the SCN without affecting the rhythmicity of the central pacemaker, indicating that the feeding–fasting cycle is a dominant zeitgeber in peripheral clocks [5]. Interestingly, body temperature also modulates self-sustaining clocks independently of the SCN [6]. The existence of these extra-SCN pacemakers that are able to coordinate clocks in peripheral tissues raised the intriguing questions of how independent these autonomous clocks actually are from the SCN, and whether this network can be maintained efficiently without central pacemaker contribution [7].

**Figure 1:**
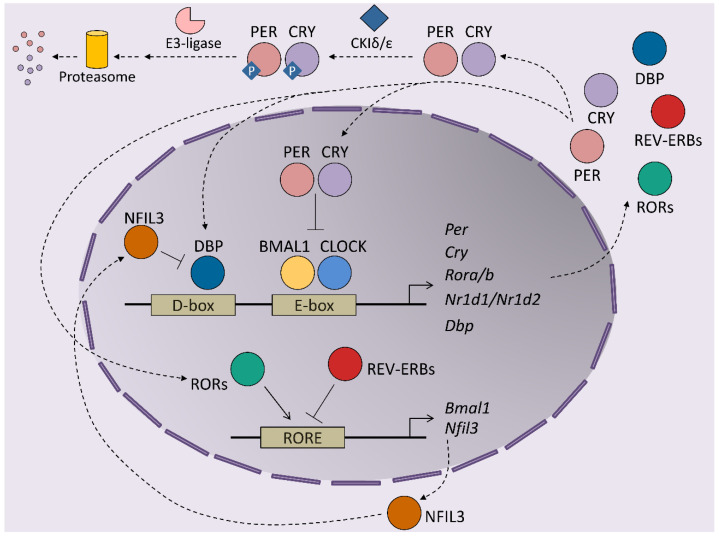
Transcriptional/translational feedback loops sustain the mammalian circadian clock. The cycle is initiated by the induction of expression of the clock genes Per, Cry, Rora/b, Nr1d1/2 and Dbp by the BMAL1-CLOCK complex. The first regulatory loop is mediated by PER and CRY proteins that translocate to the nucleus to inhibit the activity of the BMAL1-CLOCK complex, thus repressing their own expression. To restore the cycle, PER and CRY proteins in the cytoplasm are phosphorylated by CKIε/δ protein kinases to target them for degradation by the proteasome. A second loop regulates the expression of *Bmal1* by the nuclear receptors REV-ERBs (encoded by Nr1d1/2) and RORs (encoded by Rora/b), which repress and induce Bmal1 expression, respectively. Finally, in a third loop, the clock activator DBP (D-box binding protein), which induces the expression of clock component genes, is inhibited by NFIL3, whose transcription is, in turn, controlled by REV-ERBs and RORs. These three interconnected regulatory loops enable the rhythmic expression of the core clock genes and further sustain the cycling of the transcriptome.

At the molecular level, circadian rhythms are orchestrated by transcription/translation feedback loops (TTFL) of activator and repressor components of the molecular clock (Figure 1). The activator components are two bHLH-PAS proteins, BMAL1 and CLOCK, which form heterodimers that bind E-box regions on the promoters of their target genes [8]. This set of genes includes those that encode the repressor components of the clock, cryptochrome (*Cry1* and *Cry2*) and the Period proteins (*Per1*, *Per2* and *Per3*). The protein products of these genes dimerize and form complexes between themselves, which allows them to translocate into the nucleus, where they inhibit the transactivation activity of the BMAL1-CLOCK complex, thereby repressing their own expression [9,10,11]. BMAL1-CLOCK activity begins at the start of the circadian day, driving the accumulation of *CRY* and *PER* transcripts and proteins during the second part of the day. PER and CRY repressor complexes inhibit BMAL1-CLOCK activity in the nucleus during the night by repressing their own expression. As repression progresses, post-translational modifications of CRY and PER allow their inhibition and degradation by the proteasome. Most of these modifications rely on inhibitory phosphorylation by CKIε/δ and on ubiquitination by E3 ligases [12,13,14]. Turnover of the CRY and PER proteins restores BMAL1-CLOCK complexes to an active state, making them available to restart the cycle. This autoregulatory feedback loop is also maintained by the orphan nuclear receptors, REV-ERBs and RORs. Both receptors are activated by BMAL1-CLOCK and compete for binding to RORs in the promoter of their target genes, where they exert opposing roles. Namely, REV-ERBs repress BMAL1 expression, while RORs induces *Bmal1* transcription, contributing to the restarting of the clock [15,16]. This REV-ERB–ROR loop rhythm is further maintained by the DBP and NFIL3 transcription factors, which also exert activator and repressor functions, respectively, in ROR-encoding genes [17]. Post-translational regulation of the clock components is essential for maintaining the delay between the positive and negative limbs of the clock. In addition to *Cry* and *Per* post-translational regulation, phosphorylation is crucial for BMAL1 activity: phosphorylation by CKIε positively regulates BMAL1 and enhances the transcriptional activity of the BMAL1-CLOCK complex, while MAPKs phosphorylate and thereby negatively regulate BMAL1 protein [18,19]. In the particular case of the liver, around 25% of all proteins show circadian oscillation in their phosphorylation status, highlighting this post-transcriptional modification as a main timekeeper in hepatic cells [20]. In fact, this molecular oscillator not only regulates the expression of the core clock genes but also the transcription of many protein-coding genes in all tissues, thereby driving their rhythmic expression during the 24-h day cycle. Indeed, between 5 and 20% of all mouse transcripts show circadian fluctuations in any given tissue, whereby the liver is the organ that has the most fluctuations [21]. These results highlight the role of clock genes in the transmission of circadian information to non-clock proteins, integrating cellular physiology and circadian fluctuations. Indeed, the circadian output of a cell results from the interflow between the intrinsic TTFL molecular regulation of clock genes, cellular metabolism and external cues. This plasticity of the molecular clock allows cellular circadian transcriptome to adapt to these external changes [22].

The liver is probably the most widely studied peripheral clock, as disturbances of the hepatic clock negatively impact health and metabolism. There are many factors shaping the liver molecular clock and the circadian transcription/translation of genes related to different metabolic pathways. These are translated into whole-body metabolism adaptations to life—specifically, to the 24-h day cycle, food availability, temperature and nutrient demand. In this review, we will particularly focus on the different mechanisms regulating the liver clock, the circadian rhythmicity of hepatic metabolism and how the disruption of this hepatic circadian behavior can lead to the development of liver cancer through the disturbances of its metabolic function.

## 2. Modulators of Hepatic Circadian Rhythms

Liver homeostasis requires the tight circadian regulation of its metabolism, and glucose, bile acids, lipids and cholesterol are all subject to a timed circadian control [23]. This daily control of liver metabolism must adapt to changes in nutrition regimes [24,25]. The regulation of liver metabolism by feeding was demonstrated using the luciferase system in vivo. Specifically, it was shown that restricted feeding could rapidly entrain the liver circadian clock [23]. The relevance of feeding cycles for the liver clock was demonstrated by showing that feeding regimes could improve circadian gene expression patterns in mice deficient in CRY proteins [26].

The type of diet also affects the circadian liver clock; for instance, a high-fat diet (HFD) generates a profound reorganization of hepatic metabolic pathways and remodeling of the liver clock [23]. Interestingly, this reorganization of liver circadian metabolism is independent of obesity and is mediated by direct effects of the diet [25]. Recently, our group demonstrated that part of this reprogramming of liver circadian rhythm is caused by the deregulation of neutrophils infiltration in the liver, for instance from diet or jet lag [27]. Neutrophils show a circadian infiltration into the liver, where they signal hepatocytes through the secretion of the serine protease elastase, synchronizing the circadian clock and liver metabolism. However, both jet lag and unhealthy diet (for instance, deficient in methionine and choline, which leads to NAFLD, or high-fat) have been shown to disrupt the circadian neutrophil infiltration into the liver, resulting in alterations of the daily hepatic metabolism. Additionally, a ketogenic diet (KD), which is a popular high-fat, low-carbohydrate diet regime used to lose weight [28], also has profound effects on the liver clock, and especially on BMAL1-controlled genes [29].

The regulation of liver circadian rhythm is, in part, mediated by the liver-specific clock machinery, as ablation of circadian transcription genes results in dysregulation of the liver metabolic clock, including loss of oscillation in the glucose, lipid and oxidative pathways [30,31]. The intrinsic control of the liver circadian clock was demonstrated using conditional animals in which clock genes were removed only in hepatocytes. Mice lacking hepatic BMAL1 presented hypoglycemia during the day, due to an exacerbated glucose clearance, and disruption of circadian expression of glucose regulatory genes in the liver [30]. In addition, an intrinsic control of the liver clock—even in the absence of external signal or other circadian clocks—has been demonstrated using animals in which only hepatocytes express BMAL1 [32,33]. Although the key role of BMAL1 in the regulation of circadian liver metabolism is clear, it has recently been demonstrated that *Bmal1^−/−^* mice maintain circadian oscillations in their transcriptome, proteome and phosphoproteome even in the absence of any exogenous drivers, such as daily light or temperature cycles [34]. These data suggest that there are other players that keep this daily regulation. Besides this transcriptional regulation of metabolism, circulating hormones and the activation of several signaling pathways also participate in the circadian regulation [31]. The hormone-responsive nuclear receptors REV-ERBs and RORs are important liver clock components that regulate metabolism [35]. In mice, deletion of hepatic *Rev-erbs* decreases the diurnal rhythm of the clock genes *Bmal1* and *Clock* and enhances the rhythmic amplitudes of genes implicated in lipid metabolism, thereby affecting the diurnal rhythm of de novo lipogenesis [36]. Synchronization between clock genes oscillation and the expression of different enzymes and metabolic master regulators is needed to organize daily hepatic metabolism [37]. Although the circadian phases of messenger RNAs are in concordance with their output protein expression, proteomic studies have found that despite the cycling of some metabolic enzymes, their transcripts are relatively constant throughout the day [38]. In addition to circadian protein expression, circadian phosphorylation has been demonstrated to control the daily cycle of liver metabolism [20]. Moreover, acetylation and methylation are important modulators of the liver circadian rhythm. In fact, CLOCK is a histone acetyltransferase that catalyzes the acetylation of BMAL1 [39].

In the mouse liver, histone deacetylase 3 (HDAC 3) is recruited to the genome in a circadian manner. Its genomic recruitment correlates with the expression pattern of Rev-erbα, thereby regulating its lipid metabolism. Indeed, deletion of HDAC3 or Rev-erbα in mouse liver leads to hepatic steatosis [40]. Sirtuin1 (SIRT1) is another histone deacetylase that requires nicotinamide adenine dinucleotide (NAD+) as a cofactor. SIRT1 is a metabolic sensor of the energy variations in the cell as it responds to changes of NAD+/NADH ratio. The interaction between SIRT1 and BMAL1 and CLOCK proteins, at the promoters of clock-controlled genes, suggests the contribution of SIRT1 to the circadian regulation of histone lysine acetylation in the mouse liver [41]. Aging and caloric restriction have deep effects on liver circadian rhythm, and the genes that oscillate during caloric restriction are enriched on SIRT1 targets. In addition, caloric restriction potentiates cyclic protein acetylation [42].

In rodents, cholesterol and bile acid synthesis is synchronized with the timing of food intake [43]. While bile acid metabolism presents daily fluctuations in mice, in humans, cholesterol and bile acid synthesis are out-of-phase. For instance, in rodents, the rate-limiting enzyme for the synthesis of bile acids, cholesterol 7α-hydroxylase (CYP7A1), shows an early peak in the active phase [44,45]. Bile acid metabolism is, in part, regulated by several nuclear receptors, including the farnesoid-X receptor (FXR) and the liver receptor homolog 1 (LRH). FXR induces the negative co-repressor small heterodimer partner (SHP) to suppress CYP7A1 activity [23]. The clock gene *Rev*-*erbα* activates *Cyp7a1* gene expression, possibly through the inhibition of *Shp* transcription [46]. We recently demonstrated that, in mice, the liver c-Jun NH_2_-terminal kinases (JNK)1/2 have a circadian regulation in the liver [27] and control bile acid production; correspondingly, ablation of hepatic JNK1/2 results in bile acid dysregulation and cholestasis [47]. This alteration in the control of bile acid metabolism also appears in mice lacking the clock gene *Per1/2,* which present high levels of serum and liver bile acids and liver damage [48]. Overall, the crucial role of bile acids in the regulation of cholesterol metabolism prompts reconsideration of the possible contribution of bile acid circadian disruption to the hyperlipidemia found in shift workers [49].

Triglycerides, cholesterol and free fatty acids have an important circadian regulation. In fed state, ingested triglycerides arrive at the liver to be stored or utilized. During fasting, lipolysis in adipose tissue results in free fatty acids that are transported to the liver [23]. The circadian clock also controls hepatic metabolism by coordinating IRE1α-XBP1 activation, thereby modulating lipid metabolism. In addition, enzymes involved in fatty acid synthesis, such as ELOVL3, ELOVL6, and FAS, have rhythmic expression patterns [50]. For example, the circadian rhythm of ELOVL3 is impaired in *Bmal1*^–/–^ due to the reduction in the binding of REV-ERBα at the *Elovl3* promoter, as REV-ERBα is dramatically reduced in *Bmal1*^–/–^ mice [51]. Thus, clock genes play a key role in liver lipid metabolism, as the absence of a circadian clock provokes metabolic syndrome [52].

Maintenance of glucose homeostasis is a critical physiological function of the liver. Glucose uptake, gluconeogenesis and glycogenolysis must be maintained over daily periods of feeding and fasting. Daily plasma rhythms of glucagon and insulin regulate these pathways to modulate clock genes [53,54]. For example, insulin activates CLOCK expression through FOXO3; CLOCK then regulates the daily rhythm of hepatic glycogen content through transcriptional activation of glycogen synthase 2, the rate-limiting enzyme for glycogenesis [55]. CRY inhibits gluconeogenesis by repressing the glucocorticoid receptor and thereby blocking glucagon production by the pancreas [31]. Finally, BMAL1 control glycaemia by regulating GLUT2 expression; in turn, hepatic deletion of *Bmal1* induces fasting hypoglycemia, decreased liver glycogen and increased glucose clearance [30].

## 3. Circadian Rhythms and Hepatic Metabolism

The liver is a central metabolic organ that governs whole-body homeostasis, and circadian rhythms play a major role in liver homeostasis, including hepatic metabolism. In fact, over 50% of liver metabolites have a circadian rhythm that is coupled with clock genes transcription [56]. Although the liver is influenced by central and other peripheral clocks, around 10% of normally rhythmic transcripts are expressed in an autonomous manner [32]. In this circadian molecular machinery, the hepatic clock–metabolism crosstalk seems to be regulated by a circadian phosphorylation of a variety of signaling pathways involved in several metabolic processes, including the MAPK and mTOR pathways [20]. Additionally, genetic or environmental disruption of the circadian clock triggers metabolic diseases or aggravates liver pathologies, supporting the main role of clock system in hepatic metabolism.

Clock gene whole-body mutant mice show increased susceptibility to several metabolic diseases, most of which are related to lipid metabolic disorders. For example, *Clock* mutant and *Bmal1*^–/–^ mice present hyperlipidemia and hepatic steatosis [57,58,59], while *Per2*^–/–^ mice show altered lipid metabolism [60]. Lastly, *Rev-Erbα/β* ablation leads to hepatic steatosis [61]. Liver-specific mutant mice have demonstrated a central role for the liver circadian clock in many of these metabolic disorders. Indeed, liver specific ablation of *Bmal1* or *Rev-Erbα/β* show increased hepatic steatosis and dyslipidemia after HFD feeding [61,62]. Many of these mutant mice have allowed us to describe the role of the circadian clock genes in distinct hepatic metabolic processes, which are detailed below.

The circadian protein BMAL1 in the liver drives different levels and diurnal expression patterns of genes involved in lipid metabolism [63]. BMAL1 promotes de novo lipogenesis in the liver through insulin–mTORC2-AKT signaling [64]. This BMAL1-dependent lipogenic axis is needed to protect against alcoholic fatty liver disease, and mice with liver-specific *Bmal1* deletions present increased steatosis and injured liver after an ethanol-enriched diet, showing a suppression of de novo lipogenesis and fatty acid oxidation [65]. Restoring de novo lipogenesis by constitutively active AKT signaling protects against alcoholic liver disease by providing signals to the peroxisome proliferator-activated receptor (PPAR)α that mediates fatty acid oxidation [65]. It is important to note that the intracellular sensor mTOR also shows circadian oscillation that is lost in *Bmal1*-deficient livers [20]. Besides being a connector between the circadian clock and metabolism, mTOR signaling also seems to regulate central and liver clocks [66]. Inhibition of mTOR decreases the rhythm amplitude and extends the period length, disturbing the liver clock [66]. Since mTOR is a central protein that integrates nutrient and energy status to fundamental cellular processes, it is not surprising that mTOR can modify the liver clock in response to metabolic and physiological inputs.

On the other hand, the role of BMAL1 in PPARα is not restricted to the AKT-mTOR pathway. It has been shown that liver-specific deletion of *Bmal1* in mice leads to the accumulation of ROS in the liver, which increases mRNA methylation and results in the downregulation of the transcription of PPARα, thereby impacting the hepatic liver metabolism [63]. The specific ablation of *Rev-erbα* and *Rev-erbβ* in hepatocytes leads to alteration of the circadian expression of liver genes and results in the disruption of the daily rhythm of de novo lipogenesis [36]. Interestingly, treatment of obese mice with REV-ERB agonists modifies clock and metabolic gene expression, leading to reduced triglycerides and cholesterol synthesis in the liver [67]. These studies suggest that targeting the circadian clock may be a potential treatment for metabolic disorders.

Extrinsic factors, such as non-parenchymal cells, may also affect the liver circadian clock. We have recently published that neutrophils are able to control the liver circadian clock through the activation of hepatic JNKs [27]. JNKs activate *Bmal1* transcription and repress the cycling hepatokine FGF21; both events affect liver lipid metabolism, by activating lipogenesis and reducing the FGF21-metabolic regulatory effect, respectively [27]. Interestingly, JNKs have a circadian activation in mouse liver [20], and disruption of hepatic JNKs disrupts bile acid production and results in cholangiocarcinoma [47]. This circadian regulation of kinase activation might mediate crosstalk between the circadian clock and metabolism. Moreover, hepatocytes circadian rhythm is able to reprogram daily changes in liver non-parenchymal cells. In fact, hepatic REV-ERBα/β modulates rhythmic transcriptomes and metabolomes of Kupffer, stellate and endothelial cells from the liver [36]. These findings are highly relevant as the liver function has been traditionally described as being influenced by non-parenchymal cells, and this is the first report outlining that it also operates in reverse, controlling the circadian rhythms.

One of the modulators of bile acid metabolism are feeding and fasting cycles, which, at the same time, are regulated by circadian rhythms. Indeed, alteration of the circadian clock results in cholestatic disease due to bile acid metabolism dysregulation [48]. The conversion of cholesterol into bile acids is mainly catalyzed by the liver, which facilitates nutrient absorption and regulates lipid metabolism. A feedback loop constituted by ileal farnesoid X receptor (FXR), fibroblast growth factor 15 (FGF15) and small heterodimer partner 1 (SHP1) is the main regulator of bile acid synthesis [68,69]. This pathway is susceptible to regulation by the circadian machinery. In fact, the transcription factor Kruppel-like factor 15 (KLF15) regulates, in a circadian manner, the FXR-Fgf15 signaling axis, thereby controlling bile acid production [70]. Moreover, REV-ERBα contributes to this circadian regulation by controlling the accumulation of SREBP in the nucleus of hepatocytes. Accumulated nuclear SREBP induces a cyclic synthesis of cholesterols. The cholesterol-derived oxysterols serve as ligands for LXR, and cyclically activated LXR controls rhythmic *Cyp7a1* transcription, a major rate-limiting enzyme in the conversion of cholesterol to bile acids [71]. Recent results show that the circadian kinase JNK controls bile acid homeostasis through the repression of PPARα, and that repression of JNK triggers the overactivation of PPARα, thereby altering bile acid production [47]. While these findings were demonstrated in mouse models, it is important to note that in humans, bile synthesis also follows a circadian rhythm [45], and it is possible that the human homologs of these regulators may be also involved in human bile acid homeostasis.

The liver plays an important role in the regulation of glucose homeostasis. Although glucose availability is the master signal of the regulation of glucose metabolism, the central circadian clock rhythmically regulates related processes, such as insulin and glucagon secretion [53,54]. Indeed, surgical ablation of the SCN impairs the control of glucose homeostasis [72]. While the control of glucose metabolism has traditionally been linked to the central clock in the SCN, different studies, in the past, have pointed to the critical role of the liver clock in glucose homeostasis. Liver-specific deletion of *Bmal1* demonstrated that circadian disruption affects glucose homeostasis through a decrease in the glucose transporter GLUT2 during the resting phase, during which blood glucose levels must be maintained [30]. Thus, the liver clock anticipates the feeding–fasting cycle to adapt its metabolism to the systemic requirement of glucose through the circadian regulation of GLUT2. Moreover, mice carrying a lack-of-function mutant for Per2 show a decreased expression of hepatic glycogen synthase during refeeding, and increased activity of glycogen phosphorylase during fasting, with reduced fasting glycemia and an altered rhythm of glycogen accumulation in the liver [73]. Thus, Per2 seems to display a major role in the hepatic transcriptional response to feeding–fasting cycles. On the other hand, *Cry1/2* full-knockout mice present accelerated weight gain and impaired glucose metabolism and exhibit hyperinsulinemia and insulin resistance in liver and muscle [74]. This phenotype, at least in part, could be due to the role of Cry1 and Cry2 in the modulation of the activity of the cAMP response element-binding protein (Creb) in the liver limiting fasting gluconeogenesis [31]. Interestingly, hepatic overexpression of Cry1 lowers blood glucose levels and improves insulin sensitivity in insulin-resistant obese mice [31]. The anti-diabetic effect of hepatic Cry1 was also demonstrated. Notably, a rhythmic removal of Cry1 by autophagy allows glucose production, demonstrating that the presence of hepatic Cry1 can have an anti-diabetic effect [75]. Further, HFD feeding accelerates CRY1 autophagy, contributing to obesity-associated hyperglycemia [75]. Cry1 and Cry2 also seem to regulate glucose homeostasis by rhythmic repression of the genes encoding the glucocorticoid receptor and the phosphoenolpyruvate carboxykinase 1 (*Pck*1), which are master regulators of gluconeogenesis [76]. In an alternative mechanism for the anti-gluconeogenic role of Cry1 in the liver, the lipogenic transcription factor SREBP1 can activate CRY1, promoting the degradation of the nuclear FOXO1 and leading to a decrease in gluconeogenic gene expression [77]. All these studies indicate that CRY1 seems to be involved in multiple regulatory pathways controlling hepatic gluconeogenesis.

Circadian liver metabolism is also controlled by a variety of proteins and key enzymes. For instance, some hepatokines (e.g., FGF21) and adipokines (e.g., adiponectin) have rhythmic expression patterns that affect the rhythmic patterns of liver metabolism. FGF21 plays important roles in adaptation to fasting, such as lipolysis and ketogenesis, and is rhythmically expressed in the presence of the BMAL1-CLOCK complex and RORα, and strongly repressed by E4BP4 [78]. Further, adiponectin, which is expressed by the adipose tissue with a circadian rhythm, plays a fundamental role in the regulation of insulin sensitivity [79]. Indeed, daily rhythmic variations of circulating adiponectin modulate liver glucose metabolism in humans [79].

## 4. Role of Circadian Rhythms in Liver Cancer

Worldwide, liver cancer was the sixth most frequent cancer and the third most common cause of cancer deaths for both sexes, and the second most common cause in the particular case of males, in 2010 [80]. Hepatocellular carcinoma (HCC) is considered as the most common primary liver malignancy. Although it is commonly associated with chronic infection caused by hepatitis B virus (HBV), hepatitis C virus (HCV), alcoholic cirrhosis or aflatoxins [81], an increasing percentage of diagnosed cases of HCC has been suggested to be related to disruption of the circadian clock, obesity and non-alcoholic fatty liver disease (NAFLD) [82,83]. NAFLD is characterized by excessive fat accumulation that induces liver injury, inflammation and regeneration, progressing to non-alcoholic steatohepatitis (NASH), the step prior to fibrosis and cirrhosis, which are both predisposing factors of HCC [83]. Thus, several factors affecting lipid metabolism can participate in the development of HCC.

**Figure 2:**
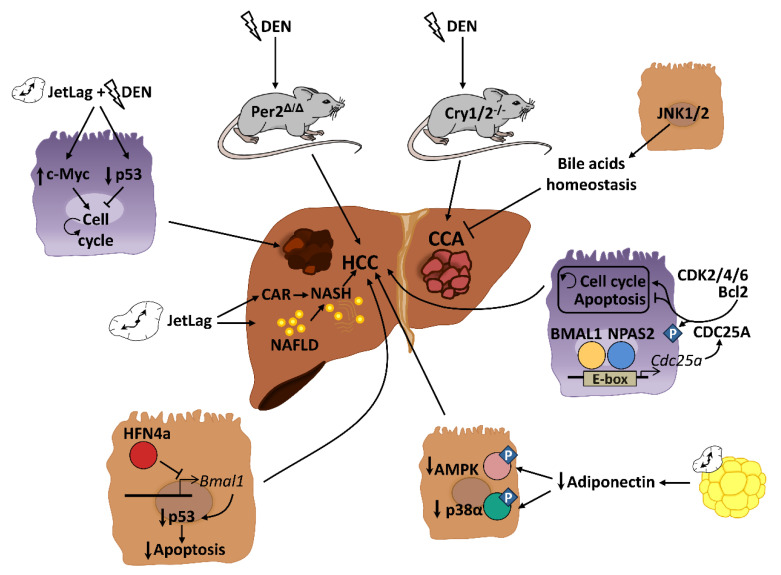
In mice, circadian disruption augments diethylnitrosamine (DEN) induced-hepatocarcinoma (HCC) by upregulating c-Myc and downregulating p53, resulting in activation of the cell cycle. Loss-of-function of *Per2* or genetic deletion of *Cry1/2* increases the frequency of DEN-induced liver cancer. In contrast, JNK1/2 protects against cholangiocarcinoma (CCA) through the maintenance of bile acid homeostasis. The neuronal PAS domain protein 2 (NPAS2) heterodimerizes with BMAL1 and induces the expression of the phosphatase CDC25A, which dephosphorylates BCL2 and cyclin dependent kinases (CDKs), thereby decreasing apoptosis and activating the cell cycle, respectively, and thus promoting HCC. Low levels of circulating adiponectin promote HCC development through a reduced activation of AMP-activated protein kinase (AMPK) and p38α in hepatocytes. HFN4a inhibits *Bmal1* expression and decreases p53 and apoptosis, positively affecting HCC development. Chronic circadian disruption induces non-alcoholic fatty liver disease (NAFLD) and spontaneous hepatocarcinogenesis through the activation of constitutive aldosterone receptor (CAR).

NAFLD and obesity are associated with people presenting alterations in sleep timing, such as night-shift workers or people with sleep dyspnea. This problem, commonly known as “social jet lag”, represents a risk factor for different metabolic disorders including cancer [84,85]. Several mouse models of circadian disruption and circadian genes knockout mouse models have allowed us to decipher the importance of circadian rhythmicity in HCC development in recent years (Figure 2). Mechanistically, circadian disruption activates the constitutive aldosterone receptor (CAR) via sympathetic nervous system dysfunction and cholestasis, and this overexpressed CAR can promote liver tumor, which drives the progression from NAFLD to NASH and, ultimately, to HCC [86]. The pro-tumorigenic effect of circadian dysfunction had been also observed in previous studies. For instance, experimentally induced chronic jet lag in mice was shown to increase the frequency of tumor formation after exposure to the carcinogenic diethylnitrosamine (DEN), in part due to an increased expression of the c-Myc oncogene and a decreased expression of the tumor suppressor p53 [87]. In addition to the potentiation of the hepatic carcinogenic effect induced by DEN, chronic jet lag also provokes a variety of hepatic tumor types in mice, such as cholangiocarcinomas, sarcomas, mixed tumors and larger tumors [88]. Remarkably, DEN itself has the ability to disrupt circadian rhythms of rest–activity and body temperature in mice [89]. DEN circadian disruption suggests that part of its carcinogen effects are mediated by alterations in the hepatic daily cycle, and that circadian reprogramming might be critical for stopping hepatic cancer development. Indeed, chronic circadian disruption also acts as a pro-tumorigenic factor in livers from steroid receptor coactivator-2 (SRC-2) knockout mice. SRC-2 works as crucial integrator of circadian behavior governed by light and metabolic homeostasis sustained by the liver; notably, a lack of SRC-2 causes mice to be unable to adapt to the stress of chronic circadian disruption and provokes NASH and HCC gene signatures [90], demonstrating the fundamental role of circadian cycles in the development of HCC.

Genetic and epigenetic alterations of clock genes can also drive carcinogenesis in different mutant and knockout mouse models. PER2, CRY1 and CRY2 seem to be critical for controlling hepatic carcinogenesis [87,89]. A lack of PER2 increases cMyc expression and disrupts clock-controlled pathways and patterns [87,89]; consequently, these mice are more susceptible to developing HCC after DEN injection [89]. Full deletion of *Cry1* and *Cry2* also disrupts the molecular circadian clock and increases chemically induced liver carcinogenesis. However, in this case, CRY seems to affect the molecular pathways involved in bile duct carcinogenesis, as its deletion dramatically increases the frequency of cholangiocarcinoma after DEN exposure [91]. These studies are in agreement with published data in humans, in which comparing HCC samples to their corresponding adjacent normal tissue shows significant alterations in the circadian genes *Per* and *Cry* [92,93,94]. As mentioned above, mice with loss-of-function of the *Clock* gene spontaneously develop NAFLD in certain chow diet conditions (such as HFD), which can progress to steatohepatitis and cirrhosis with age [58]. The additional deletion of the apolipoprotein ApoE, whose deficiency increases the susceptibility to NALFD under HFD conditions, increases the liver pathology in these mice, including the augmented expression of cancer markers in the liver [58]. Analysis of miRNA profiles in liver from *Clock* mutant mice revealed that Clock-regulated miRNAs may be involved in cancer initiation or progression by controlling genes related to cell proliferation, invasion and/or metabolism in the mouse liver [95]. Thus, deregulation of the Clock function might predispose an organism to liver cancer development. Additionally, the neuronal PAS domain protein 2 (NPAS2), a core circadian molecule analog of CLOCK, is upregulated in HCC and facilitates cancer cell survival [96]. Mechanistically, NPAS2 heterodimerizes with BMAL1 and induces the expression of the phosphatase CDC25A, which dephosphorylates the cyclin-dependent kinases (CDK)2, CDK4 and CDK6 as well as Bcl-2, thereby promoting cell proliferation and inhibiting mitochondria-dependent intrinsic apoptosis, respectively [96]. Therefore, NPAS2 is an important contributor to poor prognosis of HCC and may constitute a potential therapeutic target in HCC patients. Further, although there are no available data about the role of BMAL1 in the HCC development, it has been demonstrated that one isoform of the tumor suppressor HNF4a, which was induced in some forms of HCC, can repress *BMAL1* expression, and that forced expression of *BMAL1* in HFN4a-positive HCC prevents the growth of tumors in vivo by stimulating p53 expression and the activation of apoptosis [97].

Disruption of circadian-controlled genes that have hepato-protective functions could be a potential cause of HCC. Circulating adiponectin levels, which show circadian oscillation [79], significantly prevent HCC development through the hepatic activation of p38α and the AMP-activated protein kinase (AMPK) [98]. Decreased levels of circulating adiponectin by circadian disruption could negatively affect this antitumor activity. Similar effects could be also observed in the rhythmic disturbance of FGF-21, which is essential for preventing NAFLD progression to HCC during long-term obesogenic diet [99].

All these data indicate that a correct function of the biological clock has tumor-suppressing potential, while disturbing the normal circadian rhythm is an important risk factor for HCC. Thus, manipulation of circadian rhythms might represent an important strategy to prevent the development of HCC and to design new strategies for HCC treatment.

## 5. Conclusions

Enormous efforts have now deciphered the connections between circadian rhythms and metabolic diseases. Understanding how disruption of the liver clock contributes to liver dysfunction and promotes HCC could us help to prevent and treat these diseases. Notably, manipulation of circadian rhythms, such as through diet regimes or re-setting sleep–wake stages, might represent an important strategy for both preventing the development of HCC and designing new strategies for effective HCC treatment.
